# Binding of Carbon Monoxide to Hemoglobin in an Oxygen Environment: Force Field Development for Molecular Dynamics

**DOI:** 10.1021/acs.jctc.4c00029

**Published:** 2024-02-24

**Authors:** Mingrui Jiang, Chi-Hua Yu, Zhiping Xu, Zhao Qin

**Affiliations:** † Laboratory for Multiscale Material Modeling, Syracuse University, 151L Link Hall, Syracuse, NY 13244, USA; ‡ Department of Civil and Environmental Engineering, Syracuse University, 151L Link Hall, Syracuse, NY 13244, USA; § Department of Engineering Science, National Cheng Kung University, No.1, University Road, Tainan City 701, Taiwan; ∥ Applied Mechanics Laboratory, Department of Engineering Mechanics, Tsinghua University, Beijing 100084, China; ⊥ The BioInspired Institute, Syracuse University, Syracuse, NY 13244, USA

## Abstract

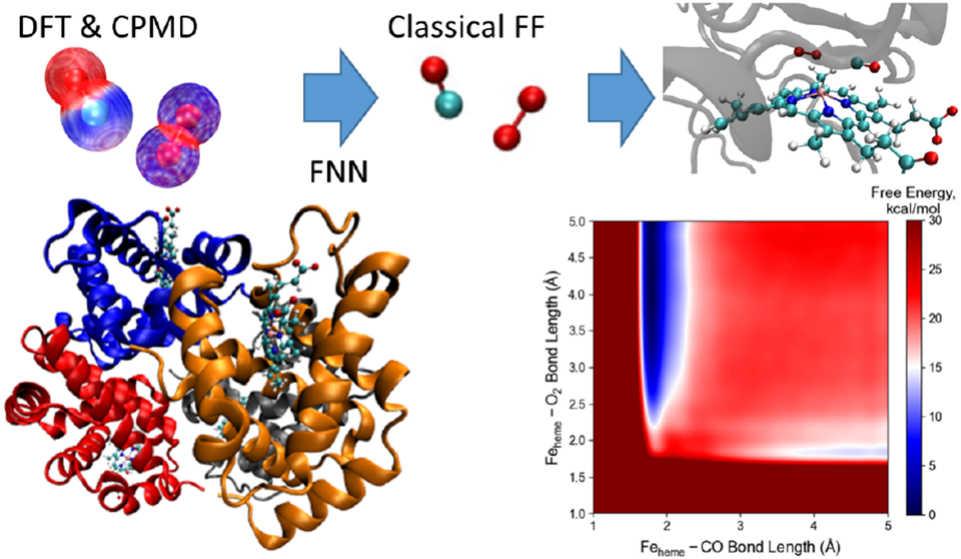

Carbon monoxide (CO) is a byproduct of the incomplete combustion of carbon-based fuels, such as wood, coal, gasoline, or natural gas. As incomplete combustion in a fire accident or in an engine, massively produced CO leads to a serious life threat because CO competes with oxygen (O_2_) binding to hemoglobin and makes people suffer from hypoxia. Although there is hyperbaric O_2_ therapy for patients with CO poisoning, the nanoscale mechanism of CO dissociation in the O_2_-rich environment is not completely understood. In this study, we construct the classical force field parameters compatible with the CHARMM for simulating the coordination interactions between hemoglobin, CO, and O_2_, and use the force field to reveal the impact of O_2_ on the binding strength between hemoglobin and CO. Density functional theory and Car–Parrinello molecular dynamics simulations are used to obtain the bond energy and equilibrium geometry, and we used machine learning enabled via a feedforward neural network model to obtain the classical force field parameters. We used steered molecular dynamics simulations with a force field to characterize the mechanical strength of the hemoglobin–CO bond before rupture under different simulated O_2_-rich environments. The results show that as O_2_ approaches the Fe^2+^ of heme at a distance smaller than ∼2.8 Å, the coordination bond between CO and Fe^2+^ is reduced to 50% bond strength in terms of the peak force observed in the rupture process. This weakening effect is also shown by the free energy landscape measured by our metadynamics simulation. Our work suggests that the O_2_-rich environment around the hemoglobin–CO bond effectively weakens the bonding, so that designing of O_2_ delivery vector to the site is helpful for alleviating CO binding, which may shed light on *de novo* drug design for CO poisoning.

## 1 Introduction

Carbon monoxide (CO) is a hazardous gas that is produced by the incomplete combustion of carbon-based fuels, such as wood, coal, gasoline, or natural gas. It is massively produced as a byproduct when there is insufficient oxygen for the complete oxidation of carbon in fuels. For example, a fire accident that is out of control, stoves and furnaces with inadequate air supply, vehicle engine exhaust, industrial emissions from steel and cement production, smoking, and cooking.^1−4^ It can quickly build up and lead to a serious toxicity to the human body as hypoxia or it can slowly cause long-term health problems or threaten life when accumulates.^2^ The normal function of the human body is enabled by cellular respiration, which requires sufficient oxygen delivery by hemoglobin, a protein found in red blood cells, with a primary function to bind to four oxygen molecules in the lungs and distribute them to body tissues. CO inhibits the oxygen (O_2_)-binding site in hemoglobin due to its higher affinity to Fe^2+^ in heme (Fe_heme_), where heme is a planar molecule centering at Fe^2+^ in the hemoglobin structure, residing in each so-called heme pocket.^5,6^ A hemoglobin bonded to CO molecules forms carboxyhemoglobin, which is a more stable complex than oxyhemoglobin as the protein bonded to O_2_,^6^ making it lack the function of O_2_ delivery. Clinically, patients with symptoms of CO poisoning are treated by hyperbaric O_2_ therapy, which supplies oxygen with a pressure usually higher than 2 atm,^7−10^ since carboxyhemoglobin can be converted into oxyhemoglobin when exposed to high O_2_ concentrations. However, the hyperbaric oxygen condition requires heavy equipments, which may not be immediately available, and thus, it is crucial to investigate other strategies of CO dissociation. There are experimental works measuring the rate constants of CO dissociation from carboxyhemoglobin in different species, and under different physical (such as light) and chemical (such as pH) conditions, providing a view of CO dissociation on a macroscale,^11−13^ and they have shown that stimuli, such as light and suitable pH range, can weaken the coordination bond effectively. It is not clear how O_2_ can be effectively used to drive the dissociation of CO from carboxyhemoglobin, which is important for designing an effective treatment of CO poisoning. However, direct experimental observations at the molecular scale of the CO binding and unbinding are extremely difficult if not impossible, making the molecular simulation crucial.

**Figure 1:**
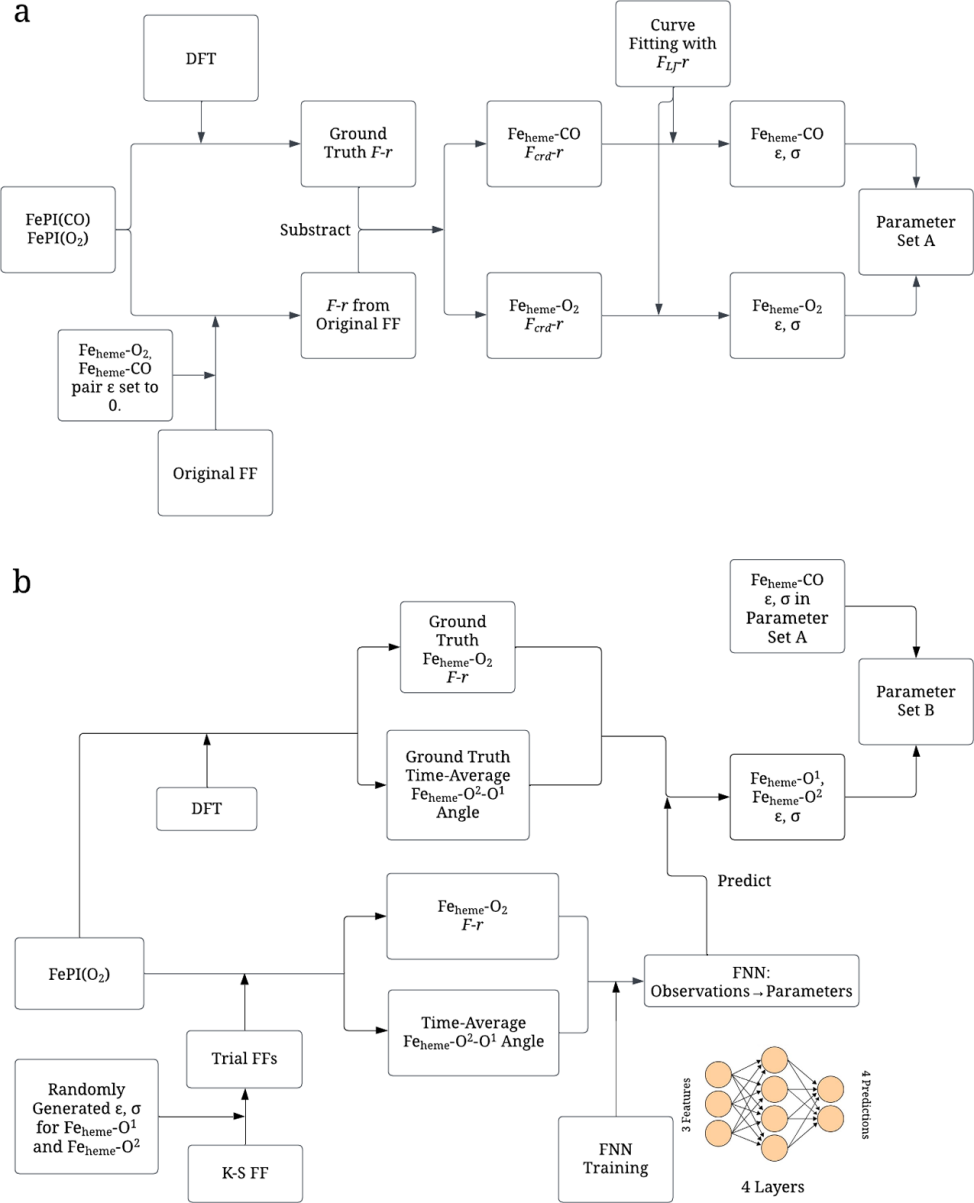
Flowchart of the parametrization procedure for (a) Parameter Set A and (b) Parameter Set B. The detailed explanation for (a) and (b) can be found in f and 2.3, respectively.

Advanced fully atomistic modeling methods^14^ make it feasible to directly simulate the dynamic rupture process of the Fe_heme_–CO coordination bond within a carboxyhemoglobin in the presence of O_2_ molecules, but there is a dilemma between the accuracy, time, and scale complexity of the model. On the one hand, quantum mechanics calculations based on modeling individual ground state electrons of the molecular system and enabled through density functional theory (DFT) calculations are applied by many studies to look into the atomic-scale details of the bonding between heme and small molecules, such as CO, NO, O_2_, and water,^15−24^ but the method is not practical to simulate the dynamics of the complicated molecular system in a water environment due to the extremely high computational demand and the uncertainty to reach numerical convergence. On the other hand, molecular dynamics (MD) based on a force field (FF) that simplifies the interatomic interaction with mathematical functions provides a feasible way to solve the problem, but its accuracy and speed highly depend on the FF. Researchers use FFs compatible with CHARMM, one of the FFs widely used in protein modeling,^25−27^ to carry out MD simulations to compute the strength of the Fe_heme_–CO bond and the conformational changes. One FF for simulations of heme and gas ligands (GLs, only for CO and O_2_ in this work) was developed by Kuczera et al.,^28^ while a three-point CO model was developed by Straub and Karplus as an improvement,^14^ designed to be applied in CHARMM FF. However, studies based on the FFs usually produce longer equilibrium distances between Fe_heme_ and CO than the results from DFT calculations.^14,23^ As coordination interaction is a multibody chemical interaction, which is much more complicated than pair interaction (e.g., vdW or hydrogen bond), specific approximation is required and parameters capable of describing the rupture of the coordination bond will need to be developed (Figure 1).

Introducing bias potentials in an MD simulation is an efficient way for sampling specific scenarios during the simulation.^29^ Bias potentials, such as harmonic springs, square wells, and Gaussian peaks, on given collective variables of the molecular system can be introduced during the simulation, and the analysis can be effectively performed with a shorter simulation timespan. The steered molecular dynamics (SMD) method, which resembles the nanoscale pulling test with an atomic force microscope, can effectively simulate bond deformation under a tensile loading force up to failure.^30,31^ Bias potential as a collection of Gaussian peaks is generally introduced in metadynamics simulations,^32−34^ which is useful to probe the energy landscape of bonding. In well-tempered metadynamics simulations, the height of the Gaussian peaks decreases with the simulation time, to guarantee the convergence of the energy landscape.

To unveil the rupture of the Fe_heme_–CO coordination bond in an O_2_-rich environment via MD simulations, we reparameterize the coordination interactions between heme and GLs with a classical FF that is compatible with CHARMM,^14,25,27^ by DFT calculations to give ground-truth Fe_heme_–GL energy and by Car–Parrinello molecular dynamics (CPMD) simulations to obtain the equilibrium Fe_heme_–O–O angle at room temperature.^35−37^ We parametrize the Fe_heme_–CO interactions by fitting the coordination potential energy landscape with a Lennard–Jones (LJ) function. We parametrize the Fe_heme_–O_2_ interactions using a feedforward neural network (FNN) model that is trained according to a series of MD simulation results for revealing the correlation between FF parameters and bond energy plus equilibrium angle. We determine the appropriate Fe_heme_–O_2_ parameters according to the energy and geometry features found in the DFT calculations and CPMD simulations. We applied this classical FF to MD simulations with SMD and metadynamics methods to explore the rupture of the Fe_heme_–CO bond in a simulated O_2_-rich environment. We found that the Fe_heme_–CO coordination bond is significantly weakened as an O_2_ molecule approaches the complex, effectively reducing both the bond strength and bond energy. Our FF parameters are compatible with CHARMM FF that can be generally used for modeling many biomolecules (e.g., amino acid, DNA, RNA, polysaccharide), which, after further careful validation, may be useful to design CO antidotes.

## 2 Parameterization of Coordination Interactions

### 2.1 Limitations of Bond Mechanics Described by Existing Type I Force Fields for CO and O_2_

**Figure 2:**
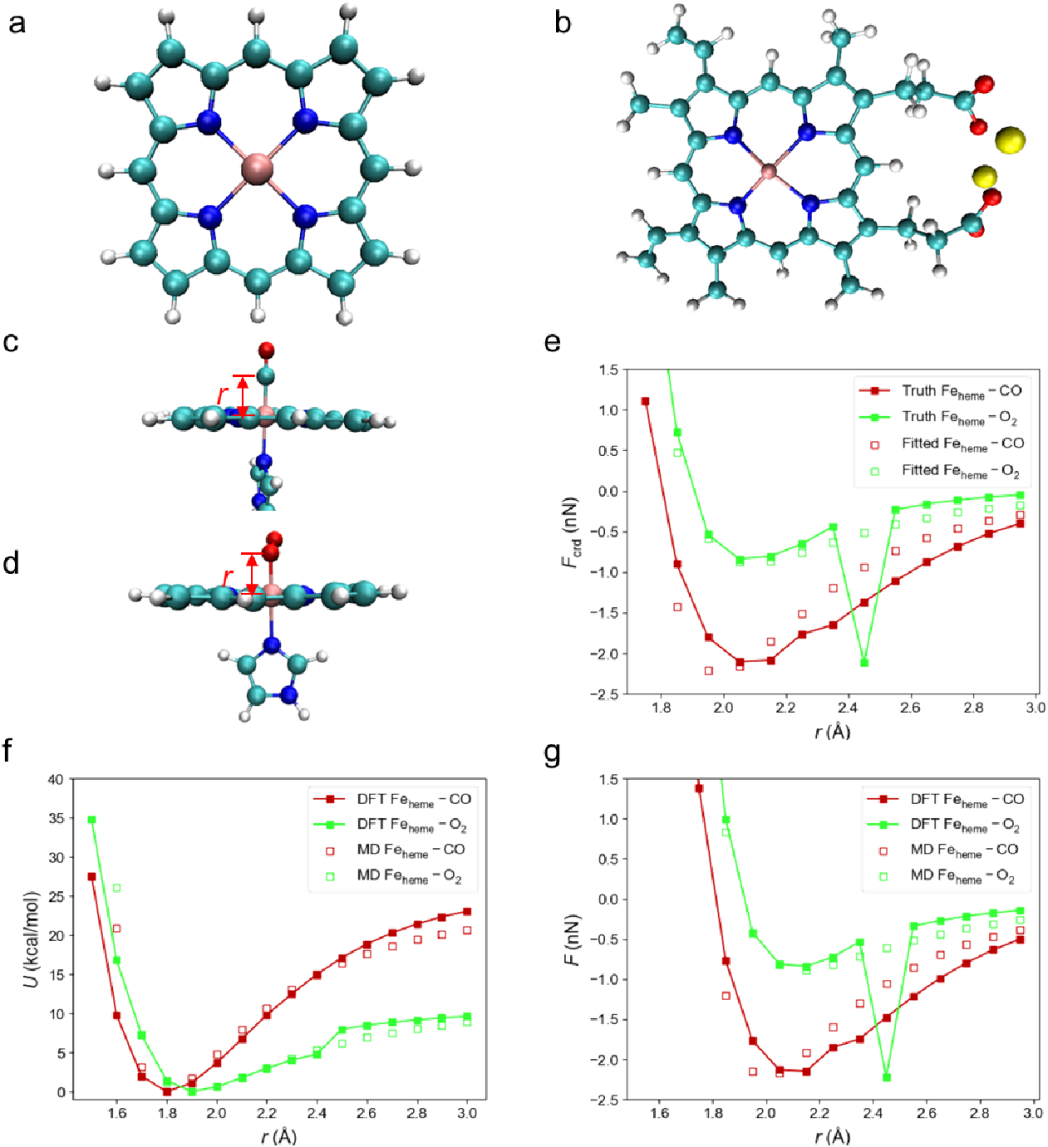
(a) The simplified and (b) complete heme structure. As the complete heme structure carries net negative charges, sodium ions are used to neutralize it. (c,d) Representations of geometries of the complexes used for evaluation and reparameterization of (c) Fe_heme_–CO and (d) Fe_heme_–O_2_ interactions with red arrows showing the varying *r*. (e) Reparametrization of the FF by fitting the LJ force (*F*_LJ_) to the *F*_crd_ obtained from subtracting the *F* obtained in zeroed K–S FF from the *F* obtained in DFT calculations. The drop at 2.45 Å is a result from the finite difference scheme to obtain *F*, as the elevation in *U* from 2.4 to 2.5 Å is greater than neighboring points. (f) The *U*–*r* curve and (g) the *F*–*r* curve produced by the Parameter Set A compared with the DFT results. The *F*_crd_*–r* and *F–r* curves are close because the noncoordination interaction is small. In all visualizations of molecular systems, carbon atoms are colored cyan, hydrogen atoms are colored white, nitrogen atoms are colored blue, oxygen atoms are colored red, iron atoms are colored pink, and sodium atoms are colored yellow.

Section 2 shows the main procedure for reparameterizing the Feheme−GL interactions (Figure 1). To find how well the mechanics of Fe_heme_-GL interactions are explained by the FF by Kuczera et al. with the Straub and Karplus three-point CO model (K–S FF),^14,28^ results from DFT calculations are used as a reference. We applied the B3LYP functional in our DFT calculations for giving ground-truth *U*-*r* curves, as the B3LYP functional is widely applied for studying heme-related molecular systems.^15−19,38^ In DFT calculations, we use a simplified structure of heme with all side groups connected to the ring structure replaced with hydrogen (Figure 2a,b), as this structure has been applied in many DFT studies of heme molecular systems.^16,17,19−21,38^ We relax the 6-membered heme–imidazole–GL (FePI(GL), where FeP is the representation of heme, alternatively iron porphyrin, I represents imidazole, and GL can be CO or O_2_) complexes in vacuum and vary the Fe_heme_–GL bond length *r* in the out-of-plane direction to find how the total potential energy *U* and the force acted on GL given by *F* = −d*U*/d*r* change, while keeping all other parts of the geometry the same (Figure 2c,d). The *F* is given by the difference between adjacent *U* values divided by the interval, which is 0.1 Å.

We evaluate the *U* and *F* using K–S FF,^14^ and we find it does not accurately represent the *U* (Figure S1a) and *F* (Figure S1b) of FePI(GL) complexes as a function of *r*, compared to the results evaluated in DFT calculations. It is shown that the electronic transfer happens when the GLs are bonded to the Fe_heme_ (Figure S2).^39^ Such electronic transfer requires careful considerations in the parametrization process. We introduce an additional pair-specific (i.e., only applicable to calculate the force between an atom pair with two specific types) LJ potential (*U*_LJ_) term to model the bonding, as the pair-specific LJ potential (defined by the NBFIX card in CHARMM FF) is the nonbonded potential that well represents the energy profile (Figure S1a) and is defined in the CHARMM FF. This function is able to describe the interaction between a specific pair of atomic species rather than describing the interaction between one atom species and all atom species (such as electrostatic potential and general LJ potential), to represent this interaction.

### 2.2 Parameterization Based on Potential–Bond Length Relationships of Complexes

We set the *ε* of the LJ potential implemented with CHARMM FFs () between Fe_heme_ and the bond-forming atoms of the GLs (carbon in CO and oxygen in O_2_) in the original FF as zero, and the LJ force (*F*_LJ_ = −d*U*_LJ_/d*r*) is used to fit for the difference produced by the DFT calculation and K–S FF results (*F*_crd_, means the contribution of coordination interactions), with *r* in the domain of 1.75 Å ≤ *r* ≤ 2.95 Å (Figure 2e). The reparameterized *ε* and σ values are summarized in Table S1 (Parameter Set A). Parameter Set A better reproduces the *U* (Figure 2f) and *F* (Figure 2g) of the complexes as a function of *r* than the original K–S FF. Both *U*–*r* and *F*–*r* curves are overlapping as much as possible, except on singularity points so that once the complexes are put into MD simulations, the evaluation of forces will be more accurate.

A potential issue with the Fe_heme_–O_2_ parameters in Parameter Set A is that the geometry of the FePI(O_2_) complex prevalent in the MD simulation with Parameter Set A is notably different from the optimized geometry in the DFT calculations (Figure S3). To take thermodynamic excitation into account, we used an *ab initio* molecular dynamics approach to finely tune the FF parameters for Fe_heme_–O_2_ interaction.

### 2.3 Parameterization of Fe_heme_–O_2_ Interactions Based on Potential–Bond Length Relationships and Dynamical Conformational Space

**Figure 3:**
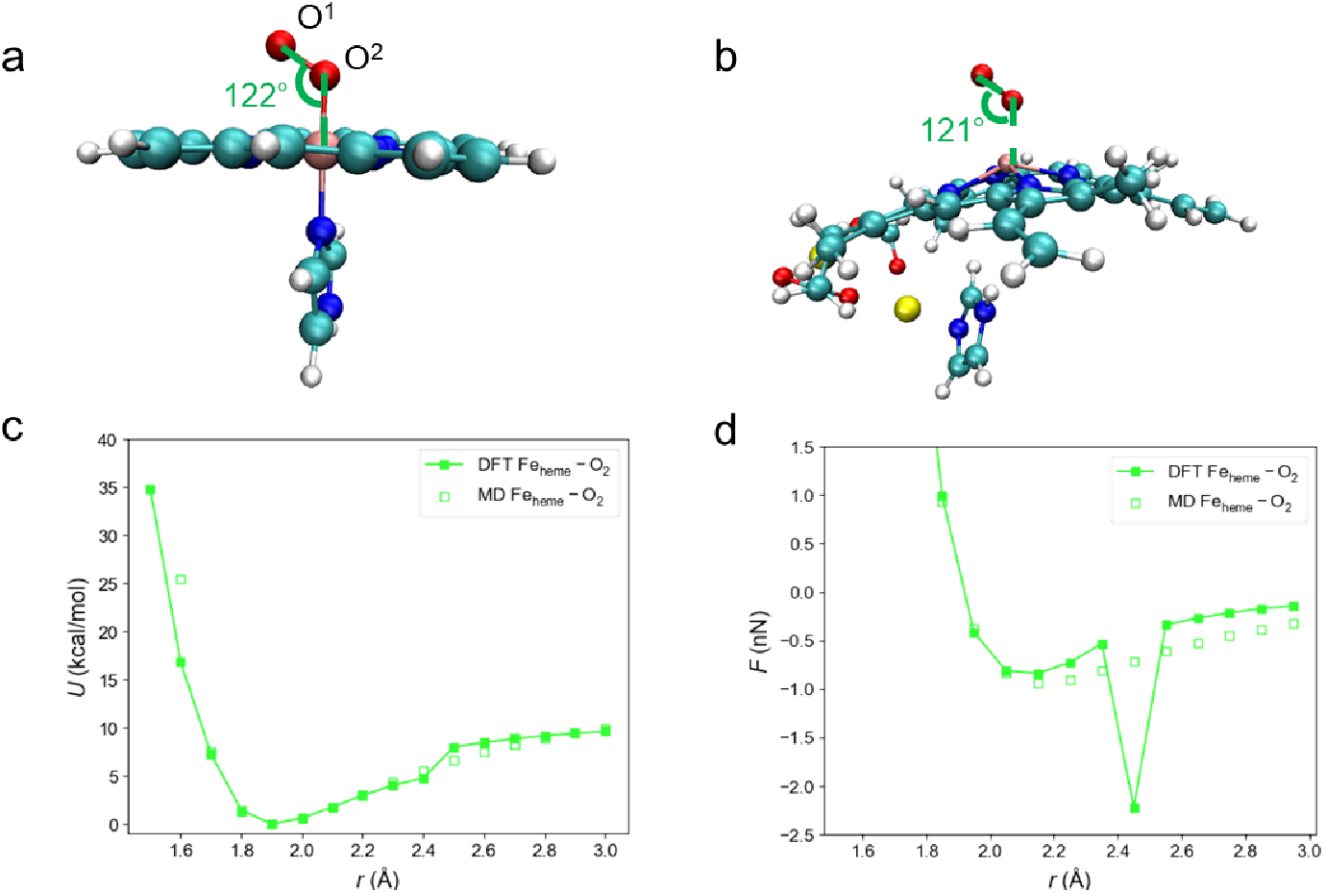
(a) The representation of labels of O^1^ and O^2^ and the Fe_heme_–O^2^–O^1^ angle. The marked value of the angle is the mean value obtained in the CPMD simulation over 20 ps. The Fe_heme_–O^2^–O^1^ angle obtained by the DFT methods can be found in Table S2. (b) An exemplary snapshot taken equilibrium MD simulations of FePI(O_2_) with Parameter Set B. The value of the angle marked on it implies the mean value over 20 ps. (c) *U*–*r* curve and (d) *F*–*r* curve for Fe_heme_–O_2_ interactions produced by the Parameter Set B compared with the DFT results.

We perform the *ab initio* molecular dynamics with CPMD,^40,41^ a DFT-based dynamics simulation technique of atomic systems on the FePI(O_2_) complex in vacuum at 300 K using the PW91 ultrasoft pseudopotential. Unless the term CPMD is used, all MD throughout the paper refers to classical FF-based MD. We label atoms in the molecule in the molecule in O_2_ as O^1^ and O^2^ accordingly. We measured the mean value of the Fe_heme_–O^2^–O^1^ angle (Figure 3a) obtained in the CPMD simulation for 20 ps on the FePI(O_2_) complex. It is shown that such a drastic difference does not exist in the CPMD simulation. The potential reason might be that only one of the oxygen atoms out of the two in the O_2_ molecule has a coordination interaction with Fe_heme_. To enforce the dynamic Fe_heme_–O^2^–O^1^ angle having a value compatible to CPMD simulation results as we construct Parameter Set B, we label O^1^ and O^2^ as two distinct atomic species with different LJ parameters when interacting with Fe_heme_. Considering this, we tried different combinations of ε and σ for Fe_heme_–O^1^ and Fe_heme_–O^2^ interactions (ε_1_, σ_1_ for Fe_heme_–O^1^, and ε_2_, σ_2_ for Fe_heme_–O^2^), getting a database for mapping from the Fe_heme_–O^2^–O^1^ angle to ε_1_, σ_1_, ε_2_, and σ_2_ values. We design an FNN model that takes the mean value of the Fe_heme_–O^2^–O^1^ angle measured in MD simulations as one of the input features. We also use the form of LJ force to directly fit the *F*–*r* curve given in the MD simulations with different combinations of ε_1_, σ_1_, ε_2_, and σ_2_ to get ε_f_ and σ_f_ as two additional features appended into the database to ensure that the Parameter Set B could produce the ground-truth *F–r* curve (see Section 3.3 for details). By feeding the 3 features measured in the CPMD simulation and fitted from DFT calculations to the tuned FNN model, the resulting ε_1_, σ_1_, ε_2_, and σ_2_ are obtained (Parameter Set B, with Fe_heme_–CO parameters the same as Parameter Set A). The mean Fe–O^2^–O^1^ angle measured in an MD simulation with Parameter Set B is in good agreement with the result in the CPMD simulations (Figure 3b). And Parameter Set B produces the *U–r* curve and *F–r* curve of Fe_heme_–O_2_ interactions well (Figure 3c,d). We summarize Parameter Set B in Table 1. For the results presented in the main text, unless specified, all of them are obtained with Parameter Set B.

**Table 1:** Parameter Set B with Fine-Tuned Parameters against CPMD

atom pair	ε, kcal/mol	σ, Å
Fe_heme_, C in CO	–21.49	1.79
Fe_heme_, O^1^	–4.37	2.74
Fe_heme_, O^2^	–6.55	1.90

## 3 Computational Details

### 3.1 DFT Calculations for Obtaining *U*–*R* Curves

We optimize the geometry of the structures, followed by translating the GL to the desired out-of-plane distances and calculating the single-point energy for the translated structures to obtain *U*–*r* curves. We apply the B3LYP functional for all calculations described in this section. The Gaussian 16 code is applied for all calculations described in this section.^42^ As experiments indicate that the ground spin state of both FePI(CO) and FePI(O_2_) complexes is singlet,^21^ we perform geometric optimizations for both complexes in the singlet form. The performance of our optimized geometries is evaluated against experimental data summarized in Table S2.^43^ 6-311G(d, p) basis sets are applied for all H, C, N, and O atoms for optimizing FePI(CO) and FePI(O_2_) complexes, while Lanl2dz basis set^44^ is applied for the Fe atom in the FePI(CO) complex,^19^ and Wachters–Hay basis set^45,46^ for the Fe atom in FePI(O_2_) complex. For single-point energy calculations, 6-311G+(d, p) basis sets are applied for H, C, N, and O atoms for FePI(CO) and FePI(O_2_) complexes, while the Lanl2dz basis set is applied for the Fe atom in the FePI(CO) complex and the Wachters–Hay basis set with diffusion functions is applied for the Fe atom in the FePI(O_2_) complex. We select the basis sets mainly based on how they can reproduce the dissociation energy measured in experiments. We roughly estimate the dissociation energy for both complexes as the single-point energy for the structure with a Fe–GL distance equal to 3.0 Å relative to the lowest value in the *U*–*r* curves. The comparisons of dissociation energies to experiments are summarized in Table S3.^16^ During the calculations of the *U*–*r* curves, singlet and quintet forms are considered for FePI(CO) complex, while singlet, triplet, and septet forms are considered for FePI(O_2_) complex. We plot the *U*–*r* curves with different fixed spins in Figure S4. Interestingly, we found that the energy minimum at the FePI(O_2_) *U*–*r* curve occurs in a triplet form, despite the experimental results showing it to be a singlet. It may be due to the limitations of the DFT functional and the initial structure we use. However, we point out that it may be a minor issue for FF parametrization, as MD simulations do not give information of the spin.

### 3.2 CPMD Simulations

The CPMD simulation of FePI(O_2_) is performed with cp.x executable in the Quantum Espresso package,^47−49^ applying PW91 functional and ultrasoft pseudopotentials built with Vanderbilt code^50^ for all atom species. The kinetic energy cutoff for wave functions is set as 20 hartree units, and the kinetic energy cutoff for charge density and potential is set as 150 hartree units. The temperature is set as 300 K. Electronic temperature is controlled by a Nosé–Hoover thermostat, while ionic temperature is controlled by rescaling. Spin polarization is enabled with unfixed total spin. The initial structure is constructed based on the geometric optimization using the pw.x executable under the same pseudopotentials, with some geometric parameters summarized in Table S2. To get better convergence of wave functions at the beginning of the simulation, we apply 1 hartree time unit (∼0.0242 fs) as the time step for 100 steps with damped dynamics for both electrons and ions. Restarting from this step, we use 0.1 fs as a time step, running a total simulated time of 20 ps. The atomic coordinates of the molecular structure are recorded at each 0.2 ps and applied for the evaluation of the Fe_heme_–O^2^–O^1^ angle.

### 3.3 The Feedforward Neural Network for Constructing Parameter Set B

The database for the mapping of 3 features to the 4 LJ parameters is constructed by randomly assigning 8 kcal/mol < ε_1_+ ε_2_ < 12 kcal/mol, 0.3 < ε_1_/ε_2_ < 0.6, 2.5 Å < σ_1_ < 3, and 1.8 Å < σ_2_ < 2.05 Å. For each set of ε_1_, σ_1_, ε_2_, and σ_2_, the same MD simulation procedure of giving the *U*–*r* curve is performed (see Section 3.4) and *F* is obtained in the same finite difference scheme, followed by using the formula  to fit for the ε_*f*_ and σ_*f*_ as the two features. The equilibrium angle is obtained as the mean value from each MD simulation of the FePI(O_2_) complex over 20 ps in 100 sample snapshots. 9,600 records of the mapping data are used as the training set, and 2,400 records are used as the testing set.

The feedforward neural network is a sequence of 4 fully connected layers, with 64 neurons in each hidden layer. Apart from the interface between the last hidden layer and the output layer, a rectified linear (ReLU) transformation is conducted between neighboring layers. During training, an adaptive momentum optimizer (adam) with learning rate 5 × 10^–4^ is applied for training 2,001 epochs. Mean squared error is used as the loss function. The number of layers and the number of neurons in each hidden layer are tuned, with performances from different model architectures summarized in Figure S5. The models are screened based on the minimized RMSE on testing set. Construction and training of the neural network is performed with the PyTorch library.^51^ The number of epochs for training is sufficient, as the loss value fluctuates near the final value. The RMSE for all 4 output parameters on training and testing sets is summarized in Table S4. The RMSEs for the parameters of the O^2^ are small, while the RMSE for the parameters of the O^1^ is higher, but the accuracy is acceptable when they are applied in MD simulations. Additionally, as the FNN model is designed specifically for obtaining Parameter Set B, the reliability can be directly verified by the mean angle in equilibrium MD simulations and *U*–*r*, and *F*–*r* curves shown in Figure 3b–d. Overfitting is not observed as we achieve close RMSEs in both training and setting set.

### 3.4 MD Simulations with FFs

All MD simulations are performed in the NAMD 2.14 package.^52^ The CHARMM36 force field is applied in all MD simulations for molecules other than GLs. Visualizations are done by VMD.^53^ The nonbonded interactions have a cutoff of 1 nm, while the switching algorithm is on if the distance between two atoms is between 8 Å and 1 nm. And the nonbonded interactions are not computed only if two atoms are connected within 3 covalent bonds. The neighbor list is updated every 10 steps, including all pairs of atoms whose distances are 1.2 nm or less. All the distances between hydrogen atoms and the atoms bonded to them cannot be changed. The full electrostatic calculations are performed every 2 steps. The time step is set to 2 fs. The temperature is 300 K with Langevin thermostatic control.

For simulations evaluating the potential energy compared against DFT results, the structure of heme is obtained by doing energy minimization on the independent heme molecule under K–S and CHARMM FF, followed by putting the Fe_heme_ into the origin of the coordinate system. The exact same internal coordinates for imidazole relative to Fe_heme_ in the DFT-optimized structures are taken to form the complex. No periodic boundary conditions are enforced, and the potential energy value given in step 0 is recorded.

The molecular system for all simulations regarding full hemoglobin structure is constructed by adding missing atoms for the crystallization structure of PDB: 1a3n (human deoxyhemoglobin), followed by adding water to make a water box with a thickness of 7 Å and neutralizing Na^+^ and/or Cl^–^ ions. GLs are added near the heme in the pocket of the A chain, and if CO and O_2_ are both present, they are on the same side of the heme plane. Periodic boundary conditions are applied for all calculations with a full hemoglobin structure. Before carrying on SMD or metadynamics calculations, each molecular system is relaxed for 1 ns. We perform our simulations with bias potentials by the PLUMED plugin of the NAMD package. During the relaxation, a square-well bias potential (command UPPER_WALLS) is placed on Fe_heme_–C (in CO) distance with a ceiling value of 1.89 Å and wall stiffness of 150 kcal/mol/Å^2^, and another harmonic potential is placed on Fe_heme_-O^2^ distance (command RESTRAINT) with an original length and stiffness equaling to the same value in the subsequent SMD simulations. In relaxations and SMD simulations, the stiffness (force constant in some literature) of the constant harmonic restraint on the Fe_heme_–O^2^ distance ranges from 1 to 10 kcal/mol/Å^2^, while the original length is fixed at zero. SMD controls are conducted using the MOVINGRESTRAINT command in the PLUMED package, with a stiffness of 10 kcal/mol/Å^2^ and an increment rate of the original length of 1 Å/ns. Metadynamics simulations are well-tempered, and they are performed by the METAD command in the PLUMED package, with the initial hill height of 8 kcal/mol, the sigma of 0.2 Å in both Fe_heme_–C (in CO) and Fe_heme_–O^2^ distances as collective variables, and the frequency of 1 ps/hill. To enable the rapid rebinding of the GLs, a square-well potential is placed between both Fe_heme_–C_CO_ and Fe_heme_–O^2^, with a ceiling distance of 5 Å and a wall stiffness of 150 kcal/mol/Å^2^.

## 4 Results

### 4.1 Mechanics of CO in a Simulated O_2_-rich Environment Subjected to Outward Pull

**Figure 4:**
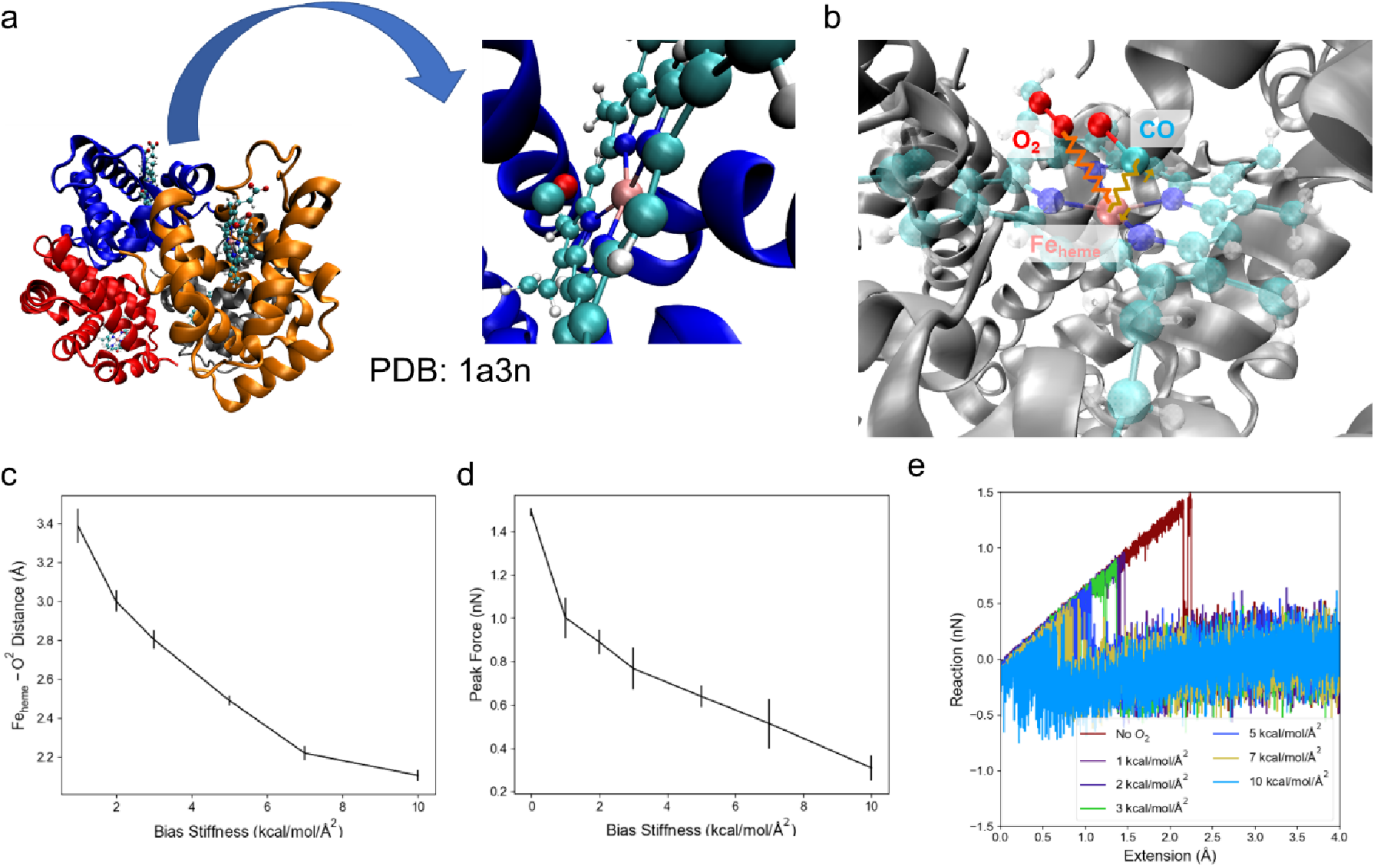
Setup and results of the SMD simulations of Fe_heme_–CO bond rupture in a simulated O_2_-rich environment with full hemoglobin structure. (a) The protein structure used for the simulation and the zoom-in view of the part with CO coordination. (b) The graphical representation of the setup of the SMD test. The orange spring represents the harmonic bias potential with a fixed original length on O_2_, and the gold spring and arrows represent the harmonic bias potential with an increasing original length on CO, which facilitates SMD simulation. (c) The average distance the constrained O_2_ molecule stays when the O_2_ is subject to the bias potential with different stiffness. Only O^2^ is taken into consideration, as it is defined as the active site for coordination interaction with Fe_heme_. Vertical lines in each data point give the standard deviation with *n* = 5. (d) Mean peak force during the rupture of the Fe_heme_–CO coordination bond with the Fe_heme_–O_2_ bias potential given different stiffness. The point with zero stiffness is the Fe_heme_–CO bond rupture force (mean value and mean value ± standard deviation, *n* = 5) given by simulations without an O_2_ molecule constrained around the Fe_heme_ to be a control group. (e) Exemplary force–extension curves given by SMD simulations without a constrained O_2_ molecule and with a constrained O_2_ molecule with different bias stiffness.

Preserving the full structure of hemoglobin (Figure 4a), we conduct SMD tests with CHARMM and K–S FF patched with Parameter Set B for the Fe_heme_–CO coordination bond in a water environment. To create a simulated O_2_-rich environment, we put an O_2_ molecule near the Fe_heme_, and added a harmonic bias potential between Fe_heme_ and O^2^ (Figure 4b).^29^ As we set the original length of the harmonic potential equal to zero, changing the stiffness will enable the O_2_ molecule to vibrate within a different radius from Fe_heme_ (Figure 4c). In the real case, since the O_2_ molecules do Brownian motions, there would be a chance for the O_2_ to stay around Fe_heme_, which is the inspiration of our original setup. Compared to the rupture force given by the Fe_heme_–CO bond without the O_2_-rich environment, when the O_2_ stays closer to Fe_heme_, the peak force during bond rupture decreases until it nearly reaches the value as though the Fe_heme_–CO bond does not exist (Figure 4d). At 3 kcal/mol/Å^2^, when the O_2_ is about 2.8 Å from Fe_heme_, the peak force becomes about half of the case without an O_2_-rich environment. It is shown that the Fe_heme_–CO bond strength is weakened to 50% of the original bond strength by the O_2_-rich environment only if an O_2_ molecule is close enough to the Fe_heme_, as ∼2.8 Å (when the bias stiffness is 3 kcal/mol/Å), and the impact of such an O_2_ molecule is significant. As this distance is around the size of the first water sphere around Fe_heme_, it practically requires that O_2_ diffuse into the water sphere to affect the Fe_heme_–CO bond strength. Therefore, the delivery of the reagent O_2_ to such a small volume will be effective to weaken and facilitate the rupture of the Fe_heme_–CO bond. The force–extension curves given by the SMD tests show that the coordination bond is brittle, and the weakening effect of the O_2_ molecule keeps the brittle property, without significant rebinding behavior of CO to Fe_heme_ (Figure 4e).

### 4.2 Free Energy Landscape for Competitive Binding Between CO and O_2_

**Figure 5:**
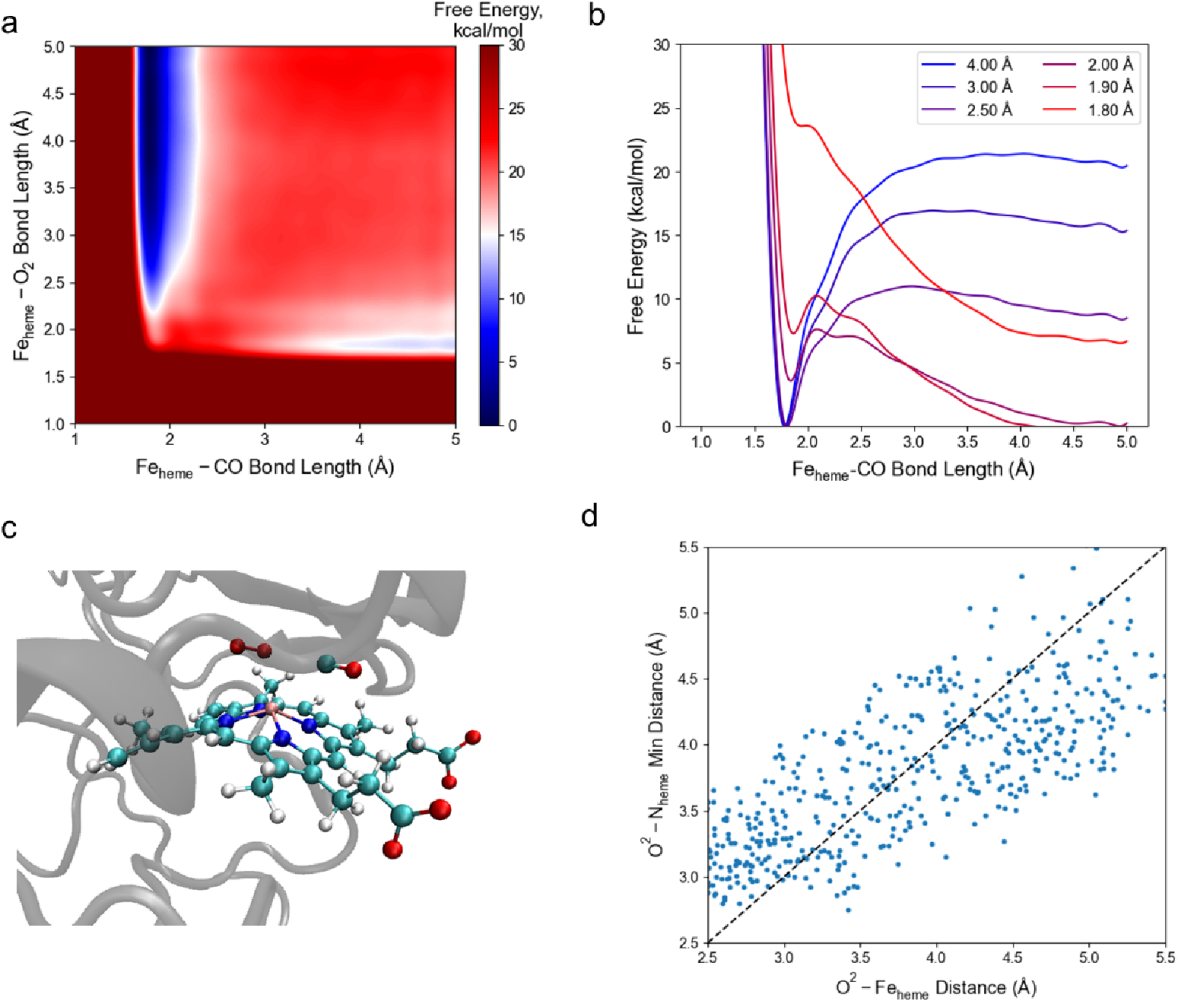
Results of the two-way metadynamics simulation. (a) The free energy landscape given by the simulation results. (b) Slices in the free energy landscape showing the free energy of the Fe_heme_–CO bond as a function of the Fe_heme_–CO bond length, with the Fe_heme_–O_2_ bond length held constant to the value indicated in the legend. (c) A snapshot showing that the Fe_heme_-CO bond is attacked by O_2_. (d) The observed pattern of the distance between the O_2_ molecule and Fe_heme_ and the distance between the O_2_ molecule and the nitrogen atoms in the heme molecule bonded to Fe_heme_ (N_heme_). The shortest distances between the O^2^ and either atom of N_heme_, and the distances between the O^2^ and Fe_heme_ are selected to plot. The dashed line shows the condition that both distances are equivalent.

To evaluate the effect of an O_2_-rich environment on Fe_heme_–CO bonding from an energy perspective, we apply a metadynamics approach in CHARMM and K–S FF patched with Parameter Set B to investigate the molecular system in which Fe_heme_–CO bond is subjected to rapid rupture and formation under O_2_-rich environment, i.e., the scenario of competitive binding. Assuming both CO and O_2_ molecules form a bond with Fe_heme_, a metadynamics MD simulation is conducted with both Fe_heme_–CO bond length and Fe_heme_–O_2_ bond length as collective variables with full hemoglobin structure in a water environment to find the free energy landscape of competitive binding of CO and O_2_ to Fe_heme_. The converged free energy landscape is shown in Figure 5a, as the energy valleys appear in the case when either the Fe_heme_–CO bond length or Fe_heme_–O_2_ bond length is approximately the equilibrium distance of the bond given by the parameters in a vacuum, which is expected. Slices with constant Fe_heme_–O_2_ bond length show that as the Fe_heme_-O_2_ bond length decreases, the effective bond energy (i.e., the maximum free energy appears from the equilibrium point of the bond to the infinity) of the Fe_heme_-CO bond becomes smaller (Figure 5b). This observation leads to the same conclusion as the last section that the Fe_heme_-CO bond is weakened, but here in terms of energy, if an O_2_ molecule approaches Fe_heme_. As shown in Figure S6, by doing single-point DFT calculations on conformations sampled from the metadynamics simulation, we find that sometimes the electronic transfer happens when the O_2_ molecule approaches the Fe_heme_–CO coordination bond. We anticipate the intensity of the electronic transfer is governed by the Fe_heme_–CO and Fe_heme_–O_2_ bond lengths, and their ratios (Table S5). It shows that with fixed Fe_heme_–CO bond length, when the Fe_heme_–O_2_ bond becomes smaller, the electronic transfer between the O_2_ and Fe_heme_ becomes more intense. Additionally, the change in the number of unpaired electrons in the FeP(CO)(O_2_) local system is also observed in the conformation with the highest ratio (i.e., Figure S6b), compared to the case analyzing FeP(CO) and O_2_ subsystems with the same coordinates. We anticipate that the mechanical effect of electron transfer is captured in our LJ parameters. We extracted and analyzed the positions of the bond-forming oxygen (O^2^) in O_2_ molecule during the metadynamics simulation, and we found that when O^2^ is sufficiently close to Fe_heme_ (<3.5 Å), it is more likely to directly diffuse toward Fe_heme_ instead of moving on the surface of the heme plane (Figure 5c,d). This highlights that the Fe_heme_–O_2_ interaction is the major interaction within a stronger effect than the interaction between O_2_ and the porphyrin ligand.

## 5 Discussion

We use the *F*–*r* curves obtained via DFT calculations as the ground truth for fit of our FF parameters. However, the authors note that the exact energy landscape will vary when different functionals, pseudopotentials, and/or basis sets are applied. Therefore, our DFT approach is mainly decided upon if our applied method can reflect experimental facts, such as optimized geometry and bond energy. We are also aware that the energy minima in FePI(O_2_) come from the triplet state, while it is singlet experimentally. It is a minor issue here, as the MD simulations do not reflect the spin state of the molecular system. Considering this, our DFT approach is sufficient for the resolution of MD.

Out of the two parameter sets we create for Fe_heme_–O_2_ interactions, Parameter Set A gives both atoms identical parameters so the chance of either atom to form a coordination bond with Fe_heme_ is not biased, whereas Parameter Set B produces better equilibrium geometry in the CPMD simulation of the FePI(O_2_) complex. Although Parameter Set B, differentiating both oxygen atoms in O_2_, weakens the chemical correspondence, the main results are not significantly changed when Parameter Set A is applied, albeit there are shifts in some numerical values (Figures S7 and S8). The free energy landscape is tilted to be slightly more favorable to O_2_ coordination than CO under Parameter Set A (Figure S8). The differentiation between O^1^ and O^2^ helps us to focus on the coordination interactions between one of them and Fe_heme_, rather than having both atoms have equal coordination interactions with Fe_heme_. In this way, Parameter Set B is better for the presentation of our main results.

The representation of the O_2_-rich environment by a harmonic bias potential can reflect the real-world situations, as the CO and O_2_ molecules do Brownian motions when they are not bonded to Fe_heme_. Therefore, conditions apply when the O_2_ molecules are occasionally present within a certain radius from Fe_heme_. By analyzing the nontrivial behaviors of the rupture of the Fe_heme_–CO coordination bond with O_2_ is present with a given geometrical constraint (provided by the bias potential), it is important for studying geometrical requirements for O_2_ to affect the Fe_heme_–CO coordination bond, giving references to drug design for targeted therapy of CO poisoning.

## 6 Conclusion

In this study, the FF parameters describing the *U*–*r* and *F*–*r* of the Fe_heme_–GL coordination bond are reconstructed, better capturing the physics for the dynamic behavior of the bonds. The FF parameters are further applied to the SMD and metadynamics simulations, revealing that the Fe_heme_–CO coordination bond is weakened only if the O_2_ bond is sufficiently close to Fe_heme_. This is consistent with the principle involved in hyperbaric O_2_ therapy in clinical practice. Our work provides FF parameters with higher reliability for heme-GL molecular systems, which can be applied in future simulations by including other biomolecules after further careful validation. Moreover, our work suggests that the O_2_-rich environment around the hemoglobin–CO bond effectively weakens the bonding, so that designing of O_2_ delivery vector to the site is helpful for alleviating CO binding, which could shed light on *de novo* drug design for CO antidotes.

## Supporting Information Available

Supplementary Figures: The *U*–*r* curve and (b) the dU/dr-r curve produced by K–S FF (labeled with MD) and DFT calculations (Figure S1); The change of electron density (Δ*n*) distributions after the formation of the coordination bond of typical complexes (Figure S2); An exemplary snapshot taken from simulations of free vibration of FePI(O2) complex in MD simulations with Parameter Set A (Figure S3); The *U*–*r* curves of (a) FePI(CO) and (b) FePI(O2) obtained in DFT calculations setting different spin multiplicity values (Figure S4); The normalized RMSEs on the testing set with different FNN model architectures (Figure S5); Changes of electronic density for conformations sampled from the metadynamics simulation (Figure S6); The mean rupture force of the Fe_heme_–CO coordination bond with different stiffness given in Fe_heme_-O2 bias potential, applying Parameter Set A (Figure S7); The results of the two-way metadynamics simulation, applying Parameter Set A (Figure S8). Supplementary Tables: Parameter Set A (Table S1); Comparison of Geometric Parameters Obtained by DFT Optimization with Experimental Findings (Table S2); Comparison of Fe_heme_-GL Bond Energy Obtained by Estimation on U-r Curves Given by DFT with Experimental Findings (Table S3); The RMSEs for four LJ parameters obtained through the FNN (Table S4); The key geometrical parameters for conformations sampled from the metadynamics simulation presented in Figure S6 (Table S5) (PDF)
Binding of Carbon Monoxide to Hemoglobin in Oxygen Environment: Force Field Development for Molecular Dynamics (Parameter Set A) (PDF)
Binding of Carbon Monoxide to Hemoglobin in Oxygen Environment: Force Field Development for Molecular Dynamics (Parameter Set B) (PDF)
