# Supplementary material for: Binding of Carbon Monoxide to Hemoglobin in an Oxygen Environment: Force Field Development for Molecular Dynamics
Source: J Chem Theory Comput. 2024 Feb 24;20(10):4229–38. doi: 10.1021/acs.jctc.4c00029 (PMC11137813; doi:10.1021/acs.jctc.4c00029)
Supplement: Supplementary file 1 — ct4c00029_si_001.pdf [file ct4c00029_si_001.pdf]

Supplementary Information

Binding of Carbon Monoxide to Hemoglobin in Oxygen Environment: Force Field Development for Molecular Dynamics

Mingrui Jiang[1,2], Chi-Hua Yu[3], Zhiping Xu[4] and Zhao Qin[1,2,5*]

1. Laboratory for Multiscale Material Modeling, Syracuse University, 151L Link Hall, Syracuse, NY, 13244, USA

2. Department of Civil and Environmental Engineering, Syracuse University, 151L Link Hall, Syracuse, NY, 13244, USA

3. Department of Engineering Science, National Cheng Kung University, No.1, University Road, Tainan City 701, Taiwan

4. Applied Mechanics Laboratory, Department of Engineering Mechanics, Tsinghua University, Beijing 100084, China

5. The BioInspired Institute, Syracuse University, NY 13244, USA

*Corresponding, E-mail: zqin02@syr.edu

Supplementary Figures

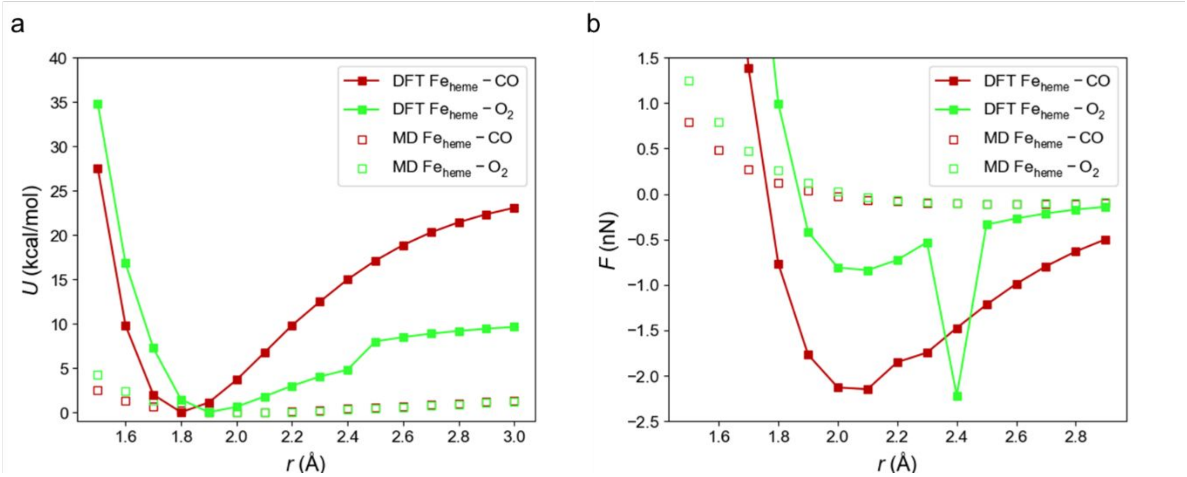

Figure S 1. (a) The *U*-*r* curve and (b) the d*U*/d*r*-*r* curve produced by K-S FF (labeled with MD) and DFT calculations.

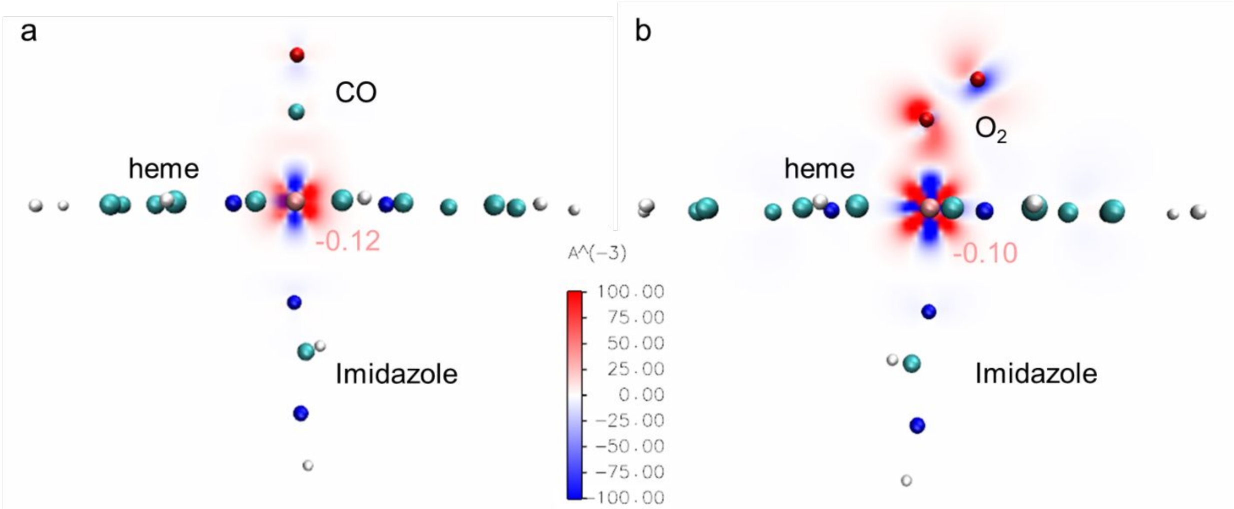

Figure S 2. The change of electron density (Δn) distributions after the formation of the coordination bond of typical complexes. The physical quantity in the legend is number of electrons per volume. Here we apply $\Delta n = n(\text{FePI(GL)}) - n(\text{FePI}) - n(\text{GL})$. The number in pink is the change of the Bader charge (unit: electron) for Fe_{heme} after the bonding with GLs.

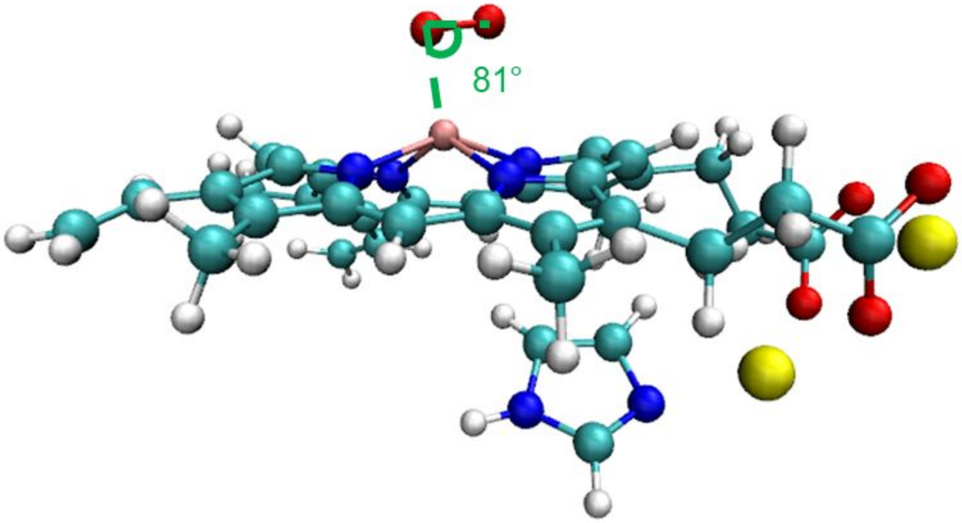

Figure S 3. An exemplary snapshot taken from simulations of free vibration of FePI(O_2) complex in MD simulations with Parameter Set A. The value of the angle marked on it implies the mean value of 20 ps.

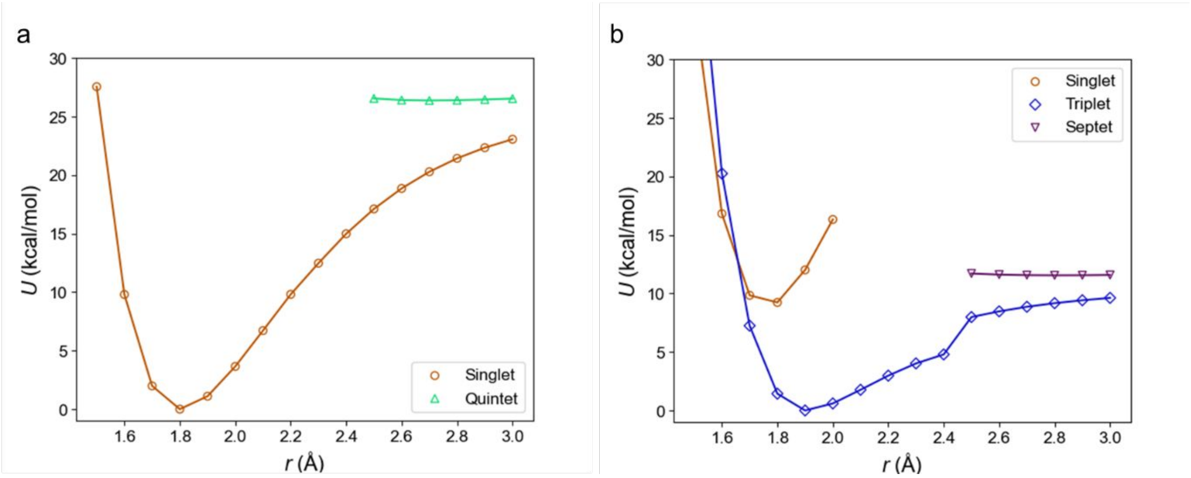

Figure S 4. The U-r curves of (a) FePI(CO) and (b) FePI(O_2) obtained in DFT calculations setting different spin multiplicity values.

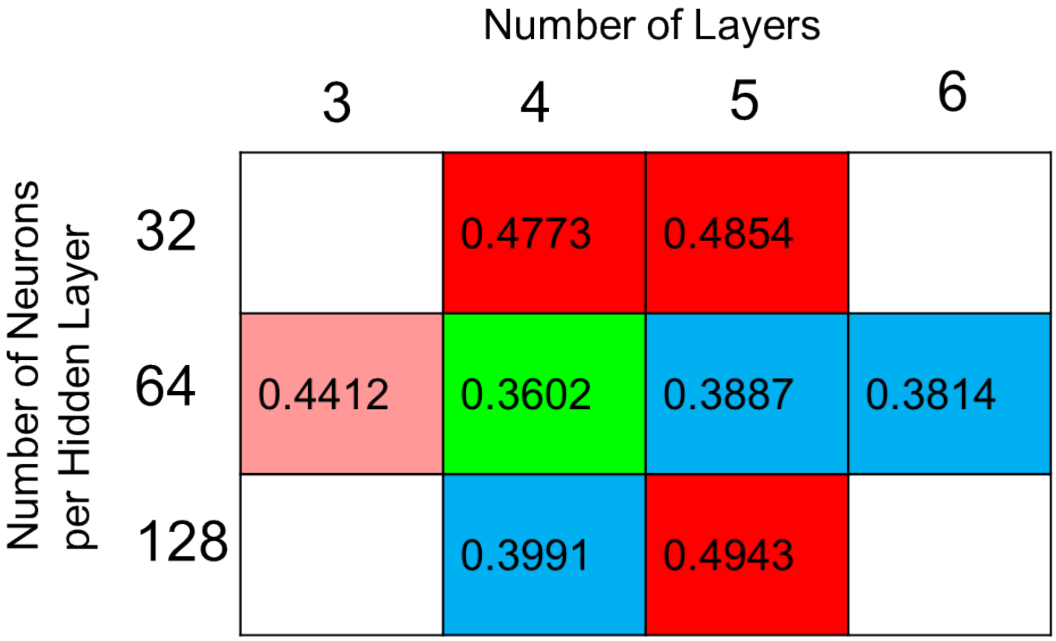

Figure S 5. The normalized RMSEs on the testing set with different FNN model architectures.

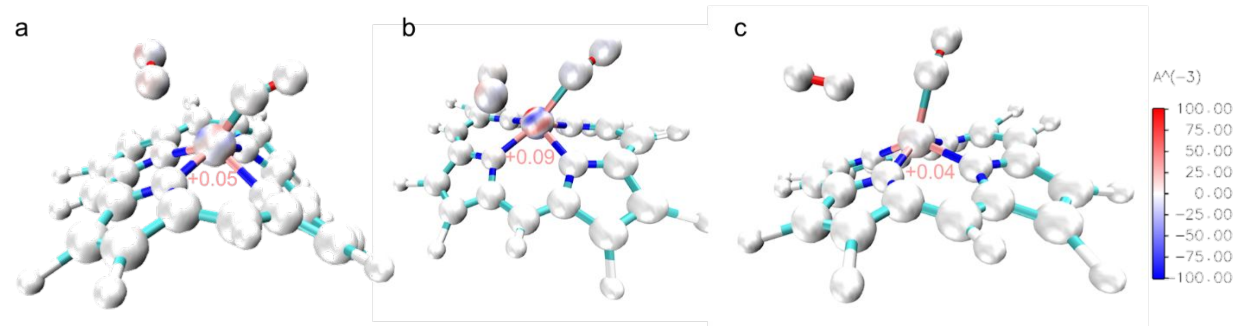

Figure S 6. Changes of electronic density for conformations sampled from the metadynamics simulation. Atomic species are indicated by the color of the bonds. All side chains are replaced with hydrogen during calculations. Here we apply $\Delta n = n(FeP(CO)(O_2))-n(FeP(CO))-n(O_2)$. The functional and basis sets used to calculate *U*-*r* curve of the FePI(CO) complex mentioned in Section 3.1 are used. All results to plot this figure apply the ground spin state. The number in pink is the change of the Bader charge (unit: electron) for Fe_{heme} when the O_2 molecule is introduced to the location.

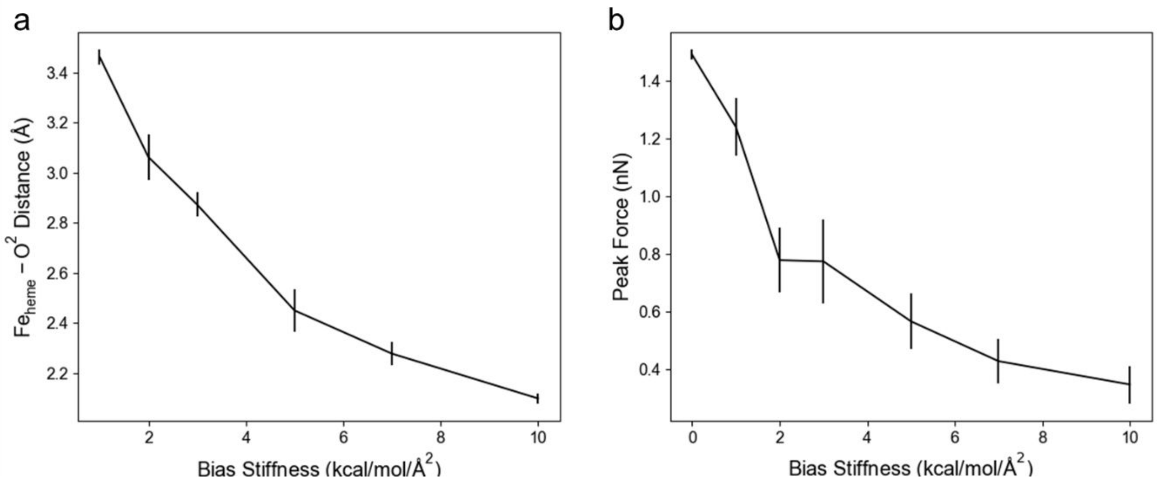

Figure S 7. The mean rupture force of the Fe_{heme}-CO coordination bond with different stiffness given in Fe_{heme}-O_2 bias potential, applying Parameter Set A. O^2 presented here means the oxygen atom to which the bias potential is added.

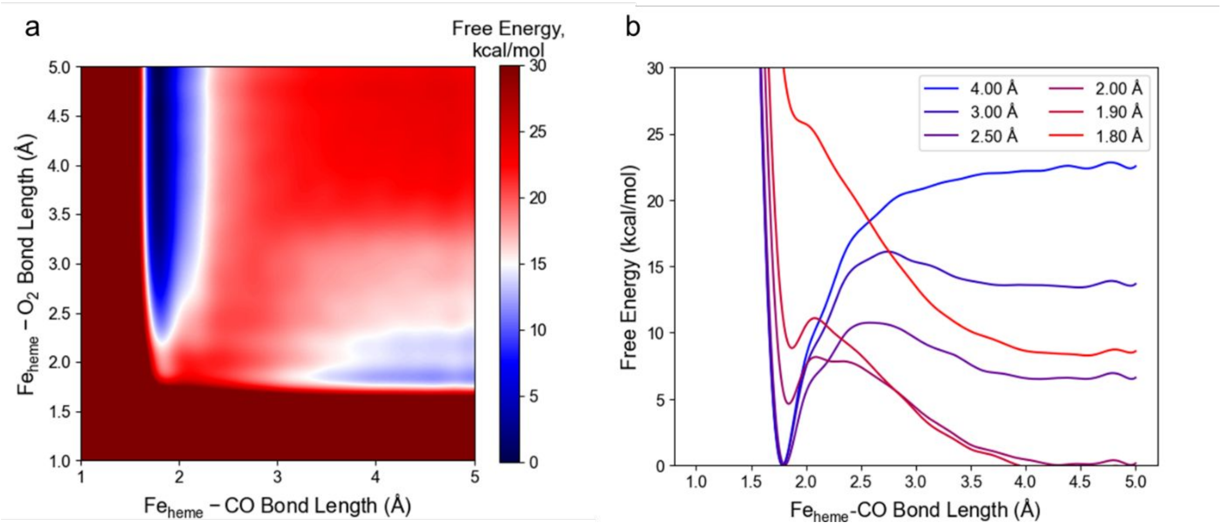

Figure S 8. The results of the two-way metadynamics simulation, applying Parameter Set A. It is shown that the energy well for Fe_{heme}-O_2 bond is deeper compared to the results given by Parameter Set A.

Supplementary Tables

Table S1. Parameter Set A

Atom Pair	$\varepsilon_{Correct}$, kcal/mol	$\sigma_{Correct}$, Å
Fe_{heme}, C in CO	Same in Parameter Set B	
Fe_{heme}, O in O_2	-7.68	1.90

Table S2. Comparison of Geometric Parameters Obtained by DFT Optimization with Experimental Findings.

Geometric Parameter	Experiment	B3LYP	PW91
FePI(CO) Complex			
Fe_{heme}-C Distance	1.82 Å*	1.81 Å	Geometry Not Used in This Work.
Fe_{heme}-N_{heme} Mean Distance	1.98 Å	2.03 Å	
Fe_{heme}-$N_{imidazole}$ Distance	2.06 Å	2.07 Å	
Fe_{heme}-C_{CO}-O_{CO} Angle	171 °	180 °	
FePI(O_2) Complex			
Fe_{heme}-O^2 Distance	1.81 Å	1.75 Å	1.75 Å
Fe_{heme}-N_{heme} Mean Distance	2.01 Å	2.02 Å	2.01 Å
Fe_{heme}-$N_{imidazole}$ Distance	2.06 Å	2.09 Å	2.09 Å
Fe_{heme}-O^2-O^1 Angle	122 °	123 °	122 °

*: All experimental data listed in this table is taken from [1].

Table S3. Comparison of Fe_{heme}-GL Bond Energy Obtained by Estimation on *U*-*r* Curves Given by DFT with Experimental Findings.

Complex	Experiment (kcal/mol)	B3LYP (kcal/mol)

FePI(CO)	19.5*	23.1
FePI(O_2)	10.1	9.6

*: All experimental data listed in this table is taken from [2].

Table S4. The RMSEs for four LJ parameters obtained through the FNN.

	ε_1 (kcal/mol)	σ_1 (Å)	ε_2 (kcal/mol)	σ_2 (Å)
Training Set	0.98	0.07	0.52	0.02
Testing Set	0.98	0.06	0.51	0.02

Table S5. The key geometrical parameters for conformations sampled from the metadynamics simulation presented in Figure S6.

Conformation	Fe_{heme}-CO Bond Length (Å)	Fe_{heme}-O_2 Bond Length (Å)	(Fe_{heme}-CO Bond Length) / (Fe_{heme}-O_2 Bond Length)
(a)	1.67	2.37	0.705
(b)	2.00	2.49	0.803
(c)	2.03	2.93	0.693

References

(1) Vojtěchovský, J.; Chu, K.; Berendzen, J.; Sweet, R. M.; Schlichting, I. Crystal Structures of Myoglobin-Ligand Complexes at near-Atomic Resolution. *Biophys. J.* **1999**. https://doi.org/10.1016/S0006-3495(99)77056-6.

(2) Radoń, M.; Pierloot, K. Binding of CO, NO, and O2 to Heme by Density Functional and Multireference Ab Initio Calculations. *J. Phys. Chem. A* **2008**. https://doi.org/10.1021/jp806075b.
