# Supplementary material for: Binding of Carbon Monoxide to Hemoglobin in an Oxygen Environment: Force Field Development for Molecular Dynamics
Source: J Chem Theory Comput. 2024 Feb 24;20(10):4229–38. doi: 10.1021/acs.jctc.4c00029 (PMC11137813; doi:10.1021/acs.jctc.4c00029)
Supplement: Supplementary file 3 — ct4c00029_si_003.pdf [file ct4c00029_si_003.pdf]

```
! Mingrui Jiang, Chi-Hua Yu, Zhiping Xu and Zhao Qin, Binding of Carbon Monoxide to Hemoglobin in Oxygen Environment:
! Force Field Development for Molecular Dynamics, JCTC, in revision
BONDS
!
!V(bond) = Kb(b - b0)**2
!
!Kb: kcal/mole/A**2
!b0: A
!
!atom type Kb          b0
!
CCO OCO    1115.0      1.128
OCO LP     0.0         0.642
O1O2 O2O2    600.000     1.2300 ! ALLOW HEM

NONBONDED nbxmod  5 atom cdiel shift vatom vdistance vswitch -
cutnb 14.0 ctofnb 12.0 ctonnb 10.0 eps 1.0 e14fac 1.0 wmin 1.5
!
!V(Lennard-Jones) = Eps,i,j[(Rmin,i,j/ri,j)**12 - 2(Rmin,i,j/ri,j)**6]
!
!epsilon: kcal/mole, Eps,i,j = sqrt(eps,i * eps,j)
!Rmin/2: A, Rmin,i,j = Rmin/2,i + Rmin/2,j
!
!atom  ignored    epsilon      Rmin/2   ignored   eps,1-4       Rmin/2,1-4
!
CCO    0.0       -0.0262       1.915
OCO    0.0       -0.1591       1.56
LP     0.0       0.0          0.0
O1O2   0.000000  -0.120000     1.700000 ! ALLOW HEM
O2O2   0.000000  -0.120000     1.700000 ! ALLOW HEM

NBFIX

FE CCO -21.49 1.79
FE O1O2 -4.37 2.74
FE O2O2 -6.55 1.90
```
